# Rod Photoreceptors Express GPR55 in the Adult Vervet Monkey Retina

**DOI:** 10.1371/journal.pone.0081080

**Published:** 2013-11-14

**Authors:** Joseph Bouskila, Pasha Javadi, Christian Casanova, Maurice Ptito, Jean-François Bouchard

**Affiliations:** 1 School of Optometry, University of Montreal, Montreal, QC, Canada; 2 Biomedical Sciences, Faculty of Medicine, University of Montreal, Montreal, QC, Canada; 3 BRAINlab, Department of Neuroscience and Pharmacology, University of Copenhagen, Copenhagen, Denmark; Dalhousie University, Canada

## Abstract

Cannabinoids exert their actions mainly through two receptors, the cannabinoid CB1 receptor (CB1R) and cannabinoid CB2 receptor (CB2R). In recent years, the G-protein coupled receptor 55 (GPR55) was suggested as a cannabinoid receptor based on its activation by anandamide and tetrahydrocannabinol. Yet, its formal classification is still a matter of debate. CB1R and CB2R expression patterns are well described for rodent and monkey retinas. In the monkey retina, CB1R has been localized in its neural (cone photoreceptor, horizontal, bipolar, amacrine and ganglion cells) and CB2R in glial components (Müller cells). The aim of this study was to determine the expression pattern of GPR55 in the monkey retina by using confocal microscopy. Our results show that GPR55 is strictly localized in the photoreceptor layer of the extrafoveal portion of the retina. Co-immunolabeling of GPR55 with rhodopsin, the photosensitive pigment in rods, revealed a clear overlap of expression throughout the rod structure with most prominent staining in the inner segments. Additionally, double-label of GPR55 with calbindin, a specific marker for cone photoreceptors in the primate retina, allowed us to exclude expression of GPR55 in cones. The labeling of GPR55 in rods was further assessed with a 3D visualization in the XZ and YZ planes thus confirming its exclusive expression in rods. These results provide data on the distribution of GPR55 in the monkey retina, different than CB1R and CB2R. The presence of GPR55 in rods suggests a function of this receptor in scotopic vision that needs to be demonstrated.

## Introduction

The *cannabis sativa* (marijuana) plant contains a group of biologically active substances, termed cannabinoids (CBs), which influence many biological functions [1], [2], including vision [3]. The CBs activate mainly two 7-transmembrane G protein-coupled receptors, the cannabinoid CB1 receptor (CB1R) that mediates most of the psychoactive effects of marijuana and the cannabinoid CB2 receptor (CB2R) that mediate the immunological effects. The persistence of cannabinoid effects in CB1R and/or CB2R knockout mice suggested the existence of additional cannabinoid receptors [4]. Following the identification and cloning of a novel human G-protein-coupled receptor 55 (GPR55), several cannabinoid ligands were shown to bind to it, suggesting that it could be a novel cannabinoid receptor [5]. Although some controversy remains, this receptor can be considered a cannabinoid receptor based on its activation by anandamide and THC, the main psychoactive compound of marijuana, at low micromolar concentrations [6]–[8]. Moreover, the endoCBs anandamide and virodhamine can modulate the activity of GPR55 [9]. However, lysophosphatidylinositol (LPI), an endogenous lipid mediator, has been described as the first ligand that potently and efficaciously activates GPR55 [6], [8], [10], [11]. In fact, the 2-arachidonoyl species of LPI may be the true natural ligand of GPR55 [12]. Agonists and antagonists of GPR55 appear to recognize different domains of the receptor corresponding to their reported pharmacological activities [13]. The atypical cannabinoid O-1602 has also been shown to act upon GPR55 [14]. GPR55 stimulation releases calcium from intracellular stores via phospholipase C [6], [8] and, in some cases, activates ERK1/2 MAP kinase [8], [11]. Interestingly, GPR55 and CB1R are capable of forming heteromers that exhibit distinct signaling properties in human embryonic kidney (HEK293) cells [15]. Additionally, GPR55 has been shown to associate with lipid rafts thus having an impact on the biological activity of this receptor [16].

GPR55 mRNA is widely distributed from moderate to low levels in the CNS, in both neuron and glia, and is also found in the vasculature and other peripheral tissues [7]. Using real-time PCR, the expression of GPR55 was found in primary microglial cells, suggesting a role for GPR55 in neuroimmunological regulation [17]. Using quantitative PCR, GPR55 mRNA expression was found in the striatum, hippocampus, forebrain, cortex, and cerebellum [18]. Human GPR55 mRNA is also strongly expressed in the basal ganglia (striatum, caudate nucleus, and putamen), moderately in the nucleus accumbens, hypothalamus, and hippocampus, and weakly in the cerebellum [19]. While the overall human to mouse amino acid sequence similarity is 97% for CB1R, and 79% for CB2R, the human GPR55 protein sequence is only 74% identical to the mouse GPR55. Nevertheless, even though the immunohistochemical localization of GPR55 in the CNS is limited, it has been found in mouse dorsal root ganglia [6]. Interestingly, the GPR55 KO mouse develops normally, no defects in brain structures are detected, and the abundance of endocannabinoids and related lipids are not affected [18]. While GPR55 appears to satisfy the criteria of a cannabinoid receptor, its pharmacology is inconsistent with several of the non-CB1R/non-CB2R effects. Thus, additional cannabinoid receptors clearly remain to be identified.

Expression patterns of CB1R and CB2R have been both localized in the vervet monkey retina [20], [21]. There are numerous evidences that show that cannabinoids have many visual effects [3] but no data are available on the expression and role of GPR55. Cannabidiol, a bioactive compound of the plant *cannabis sativa* without psychotropic effects, has been shown to bind to GPR55 with very low binding capacity on CB1R and CB2R. Moreover, cannabidiol seems to have protective effects on retinal neurons [22]. A recent study demonstrated that knocking down the expression of GPR55 with specific shRNAs partially blocked the PEA-induced increase in aqueous humor outflow facility [23]. Although a complicated cannabinoid profile has prevented its classification as a cannabinoid receptor, the therapeutic potential of GPR55 cannot be denied [24]. The present study investigates the presence of GPR55 in the monkey retina, compares the spatial expression of GPR55 to that of CB1R and CB2R, and proposes a putative role for GPR55 in retinal functions.

## Materials and Methods

### Choice of species

Monkey tissue, the experimental model for the current study, was chosen for several reasons. First, using monkey tissue allows us to infer more easily on what is really present in humans. Additionally, the anatomical similarity between the monkey and human retina is remarkable. Primates are mammals that have a macular/foveal region and multiple cone types responsible for high visual acuity and color vision. Finally, the high cross-reactivity between human and monkey antigens increases chances of success for targeting GPR55 in monkeys using an antibody directed against human GPR55 epitope.

### Animal Preparation

Six 42 months-old vervet monkeys (*Chlorocebus sabaeus*) were included in this study: three were used for the immunochemistry protocols, and fresh specimens of retina, visual cortex, and cerebellum were collected from three others for immunoblotting. The animals were born and raised in enriched environments in the laboratories of the Behavioural Sciences Foundation (St-Kitts, West Indies) that is recognized by the Canadian Council on Animal Care (CCAC). The animals were fed with primate chow (Harlan Teklad High Protein Monkey Diet; Harlan Teklad, Madison, WI) and fresh local fruits, with water available ad libitum. Infant vervets are born into an outdoor social group comprising several females, one male and other offspring of the same general age. Infants live with their parents until about 8 months of age, at which time they move to a playpen with 5 other age-mates. The natal cage is equipped with swings, perches, hiding places and jungle gyms. We do put in toys, but the animals are so busy playing with one another that they ignore the toys. In the smaller playpens, there are also swings, perches and climbing spots, as well as puzzle feeders and foraging boards. At about 18 months of age, youngsters graduate to a large, outdoor peer group of about 16 animals (like-ages, both sexes) where there are tunnels, swings, ladders, jungle-gyms and a variety of manipulanda (more complex puzzle feeders; natural forage opportunities, such as brush and vines; foraging boards). Plastic chain and baited balls are popular toys, but vervets of this age are uninterested in most other commercially available toys. The experimental protocol was reviewed and approved by the local Animal Care and Use Committee (Université de Montréal) and the Institutional Review Board of the Behavioural Science Foundation. Each animal was sedated with ketamine (10 mg/kg, i.m.), deeply anaesthetized with sodium pentobarbital (25 mg/kg, i.v.) and perfused transcardially with phosphate buffer saline (PBS pH 7.4), followed by 4% paraformaldehyde.

### Antibody characterization

All the primary antibodies used in this work, their sources and working dilutions, are summarized in Table 1. These antibodies were successfully used in previous studies and are well characterized in regards to the specific primate retinal cell type immunostaining, as described below for each antibody.

**Table 1:** Primary antibodies used in this study.

Antibody^1^	Immunogen	Source^2^	Working dilution
Rhodopsin	Bovine rhodopsin	Abcam, Toronto, ON, ab98887, Mouse monoclonal, Clone Rho 4D2	H: 1∶500
CB	Purified bovine kidney calbindin-D28K	Sigma, St. Louis, MO, C9848, Mouse monoclonal, Clone CB-955	H: 1∶250
GS	Full protein purified from sheep brain	Chemicon, Temecula, CA, MAB302, Mouse monoclonal, Clone GS-6	H: 1∶500
PKCα	Peptide mapping the aa 296–317 of human PKCα	Santa Cruz Biotechnology, Santa Cruz, CA, sc-8393, Mouse monoclonal, Clone H-7	H: 1∶500
PV	Full protein purified from frog muscle	Sigma, St. Louis, MO, P3088, Mouse monoclonal, Clone PARV-19	H: 1∶250
CB1R	Fusion protein containing aa 1–77 of rat CB1R	Sigma, St. Louis, MO, C1233, Rabbit polyclonal	H: 1∶150
CB2R	Synthetic peptide from aa 20–33 of human CB2R	Cayman Chemical, Ann Arbor, MI, 101550, Rabbit polyclonal	H: 1∶150
GPR55	Synthetic peptide from aa 207–219 of human GPR55	Cayman Chemical, Ann Arbor, MI, 10224, Rabbit polyclonal	H: 1∶200 W: 1∶500
GAPDH	The full-length rabbit muscle GAPDH protein	Sigma, St. Louis, MO, G8795, Mouse monoclonal, Clone GAPDH-71.1	W: 1∶20,000

1 Abbreviations: CB, calbindin; GS, glutamine synthetase; PKCα, protein kinase C (α isoform); PV, parvalbumin; CB1R, cannabinoid receptor type 1; CB2R, cannabinoid receptor type 2; GPR55, G-protein coupled receptor 55; GAPDH, glyceraldehyde-3-phosphate dehydrogenase; aa, amino acids; H, immunohistochemistry; W, western blot.

2 The source column indicates the commercial company, catalog reference and origin. The clone designation is given for monoclonal antibodies.

#### Rhodopsin

The mouse monoclonal (IgG1) to rhodopsin from Abcam (Cambridge, MA) was obtained by using as immunogen the bovine rhodopsin. This antibody recognizes a 39 kDa band on Western Blots and is predicted to react with human retinal tissues (manufacturer's data sheet). It has been proven effective to specifically label rods in the rodent retina [25].

#### CB

The mouse monoclonal (IgG1) to calbindin (CB) from Sigma (St. Louis, MO) was obtained by using as immunogen purified bovine kidney Calbindin-D-28K. This antibody recognizes a 28 kDa band on Western Blots. Immunostaining against calbindin is known to label cones outside the foveal region, cone bipolar cells and a subset of horizontal cells on human and monkey retinal sections [20], [21], [26]–[30].

#### GS

The mouse monoclonal to glutamine synthetase (GS) was obtained from Chemicon International (Temecula, CA) by using as immunogen the GS purified from sheep brain. This antibody generates a single 45 kDa band in adult retinal tissue [31]. This antibody labels Müller cells in rat [32]–[34] and monkey [20], [21], [35] retinas.

#### PKCα

The mouse monoclonal (IgG2a) to protein kinase C (PKC) was obtained from Santa Cruz Biotechnology (Santa Cruz, CA) by using as immunogen purified bovine PKC. The epitope is mapped to PKC hinge region (amino acids 296–317). It detects the PKCα isoform, a well-known specific marker for rod bipolar cells [36]. As stated by the manufacturer, this antibody gives a single band of 80 kDa on Western blots of human cell lines, and has been previously used for immunohistochemistry on rodent [33], [34] and monkey [20], [21], [29], [37] retinas.

#### PV

The mouse monoclonal (IgG1) to parvalbumin (PV) was obtained from Sigma (St. Louis, MO) by using as immunogen purified frog muscle PV. It recognizes a 12 kDa band from human, bovine, pig, canine, feline, rabbit, rat, and fish tissues (manufacturer's technical information). The pattern of labeling with this antibody was the same as reported previously [28], [38]. This small calcium-binding protein is expressed in the primate retina by horizontal cells [39] and the antiserum has been used to visualize monkey thalamic nuclei [40] and vervet monkey horizontal cells [21].

#### GPR55

The rabbit anti-GPR55 was obtained from Cayman Chemical (Ann Arbor, MI) by using as immunogen a synthetic peptide corresponding to the amino acids 207–219 (ILLGRRDHTQDWV) of the human GPR55 sequence. This antibody recognizes a band at 37 kDa (manufacturer's data sheet, 10224). This antibody was characterized and used to detect GPR55 expression in human trabecular meshwork cells [23].

#### CB1R

The rabbit anti-CB1R was obtained from Sigma (St. Louis, MO) by using a highly purified fusion protein containing the first 77 amino acid residues of the rat CB1R as the immunogen. It recognizes a major band of 60 kDa and less intense bands of 23, 72, and 180 kDa (manufacturer's data sheet, C1233). This antibody targets the rat CB1R but specifically recognizes the CB1R (60 kDa) from many species, including vervet monkey retinal tissue [20], [21].

#### CB2R

The rabbit anti-CB2R was purchased from Cayman Chemical (Ann Arbor, MI) and was developed by using a synthetic peptide corresponding to the amino acids 20–33 (NPMKDYMILSGPQK) of the human CB2R sequence conjugated to KLH as immunogen. This antibody recognizes a band at 45 kDa and a band at 39–40 kDa (manufacturer's data sheet, 101550). This antibody was used in human nervous tissues [41], [42] and to detect CB2R from vervet monkey retinal tissue [21].

#### GAPDH

The mouse monoclonal to GAPDH (clone GAPDH-71.1) was obtained from Sigma (St. Louis, MO) by using as immunogen purified rabbit muscle GAPDH (whole molecule). As stated by the manufacturer, this antibody recognizes monkey GAPDH and gives a single band at about 37 kDa.

### GPR55 blocking peptide

The GPR55 blocking peptide containing the human GPR55 amino acid sequence 207–219 (ILLGRRDHTQDWV; Cayman Chemical; catalog number 10225) was used in the present study for immunohistochemistry and western blot analysis. The specificity of the GPR55 antibody was also tested by preincubation with the corresponding blocking peptide. For preadsorption, 20 µg of primary antibody was mixed with 20 µg of blocking peptide for 2 hours at room temperature with occasional inversion. We then diluted that mixture 1∶200 making a final concentration of 10 µg/ml of preadsorbed antibody. The antibody-blocking peptide solution was added to the slices or blots and subsequent immunohistochemistry and western blot analysis followed the protocol as described further.

### Tissue preparation

The eyes were extracted and the retina was dissected free from the eyecup in a PBS bath. The retina was laid flat so that the vitreous body could be removed by blotting with filter paper and gentle brushing [43]. Samples of retina (4 mm^2^) were taken from the extrafoveal retina (between 1 and 5 degrees of perimetric angle), middle retina (between 6 and 25 degrees of perimetric angle) and periphery (above 25 degrees of perimetric angle). Each sample was then cryoprotected in 30% sucrose overnight and embedded in Shandon embedding media at −65°C. Retinal samples were then sectioned in a cryostat (18 µm) and mounted onto gelatinized glass microscope slides, air-dried and stored at −20°C for further processing.

### Western blotting

In order to test the specificity of our antibodies directed against GPR55, Western blots were performed on a piece of monkey tissue. A fresh dissected sample of retina, visual cortex, and cerebellum was homogenized by hand using a sterile pestle in RIPA buffer (150 mM NaCl, 20 mM Tris, pH 8.0, 1% NP-40 (USB Corp., Cleveland, OH, USA), 0.1% SDS, 1 mM EDTA), supplemented with a protease inhibitor mixture (aprotinin (1∶1,000), leupeptin (1∶1,000), pepstatin (1∶1,000) and phenylmethylsulfonyl fluoride (0.2 mg/ml); Roche Applied Science, Laval, QC, Canada). Samples were then centrifuged at 4°C for 10 minutes, and the supernatant was extracted and stored at −20°C until further processing. Protein content was equalized by using a Thermo Scientific Pierce BCA Protein Assay Kit (Fisher Scientific, Ottawa, ON, Canada). Thirty micrograms of protein/sample of the homogenate was resolved with 10% sodium dodecyl sulfate (SDS)-polyacrylamide gel electrophoresis, transferred onto a nitrocellulose membrane filter (BioTrace NT, Life Sciences, Pall, Pensacola, FL), blocked for 1 hour in 5% skim milk (Carnation, Markham, ON, Canada) in TBST (0.15 M NaCl, 25 mM Tris-HCl, 25 mM Tris, 0.5% Tween- 20), and incubated overnight with a primary antibody, namely, rabbit anti-GPR55 (1∶500) in blocking solution. The following day, the blot was exposed to a secondary antibody conjugated to horseradish peroxidase (1∶5,000; Jackson ImmunoResearch, West Grove, PA) in blocking solution for 2 hours. Detection was carried out by using homemade ECL Western blotting detection reagents (final concentrations: 2.50 mM luminol, 0.4 mM p-coumaric acid, 0.1 M Tris-HCl pH 8.5, 0.018% H_2_O_2_). The membrane was then air-stripped, reblocked, and exposed to a second primary antibody, namely mouse anti-GAPDH (1∶20,000), until all proteins of interest were tested.

### Immunohistochemistry

Single- and double-labeling of the retina were performed according to previously published methods [20], [21]. Briefly, sections were postfixed for 5 minutes in 70% ethanol, rinsed 3×5 minutes in 0.1 M Tris buffer, pH 7.4/0.03% Triton and blocked for 90 minutes in 10% normal goat serum (NDS) in 0.1 M Tris buffer/0.5% Triton. Sections were incubated overnight at room temperature with primary antibody in blocking solution. The GPR55 antibody was used conjointly with a known specific retinal cell type marker: rhodopsin, calbindin, glutamine synthetase, PKCα, or PV (Table 1). The next day, sections were washed for 10 minutes and 2×5 minutes in 0.1 M Tris/0.03% Triton, blocked in 10% NDS, 0.1 M Tris/0.5% Triton for 30 minutes and incubated with secondary antibody for 1 hour: Alexa 488 donkey anti-mouse, and biotinylated donkey anti-rabbit followed by the addition of streptavidin-Alexa 647 (1∶200), all in a blocking solution as described above. Sections were washed again in Tris buffer, counterstained with bisbenzimide (Hoechst 33258, Sigma, 2.5 µg/mL), a fluorescent nuclear marker, and coverslipped with Fluoromount-G™ Mounting Medium (SouthernBiotech, Birmingham, AL).

### Confocal microscopy

Fluorescence was detected with a Leica TCS SP2 confocal laser-scanning microscope (Leica Microsystems, Exton, PA), using a 40X (n.a: 1.25–0.75) or a 100X (n.a: 1.40–0.7) objective. Images were obtained sequentially from the green and far-red channels on optical slices of less than 0.9 µm of thickness. When co-expression of GPR55 and retinal cell markers was ambiguous in some retinal layers, co-labeling or its absence was demonstrated by taking z-stacks with optimized steps. This allowed for visualization of the cells in the X-Y, X-Z and Y-Z axes, thereby confirming the presence or absence of GPR55 in specific retinal cells. All photomicrograph adjustments, including size, color, brightness, and contrast were done with Adobe Photoshop (CS5, Adobe Systems, San Jose, CA) and then exported to Adobe InDesign (CS5, Adobe Systems, San Jose, CA), where the final figure layout was completed. The schematic panels were created using Adobe Illustrator (CS5, Adobe Systems, San Jose, CA).

## Results

### Expression profile of GPR55 in monkey retina

**Figure 1:**
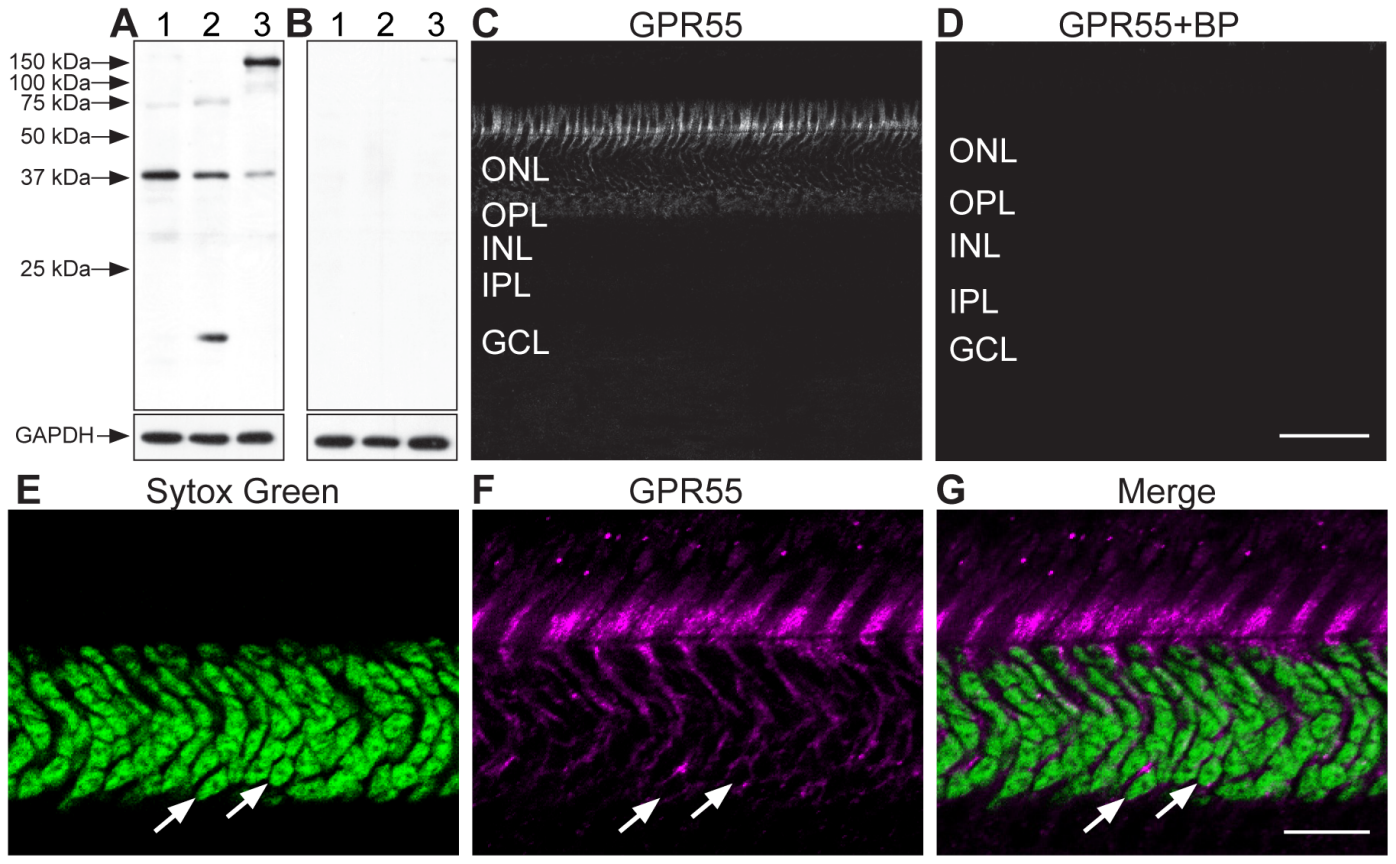
Characterization of GPR55 antibody in the vervet monkey. Western blot analysis of total protein samples from retina (**A – lane 1**), visual cortex (**A – lane 2**) and cerebellum (**A – lane 3**) showing detection of the expected protein band at 37 kDa. The band was not detected when the antibody was pre-incubated with the corresponding GPR55 blocking peptide (BP) (**B – lanes 1–3**). All lanes contained 30 µg of total protein. The lower blots for GPR55 and GPR55-BP show the expression of the protein GAPDH and demonstrates equal loading in all lanes. Immunohistochemistry on vervet retinal tissue with the anti-GPR55 antibody revealed a unique staining profile (**C**). When the GPR55 antibody was pre-incubated with its BP, it revealed an absence of staining (**D**). Double-label of Sytox (green) and GPR55 (magenta) indicated that GPR55 was not present in the nuclei of rods (**E–G**, arrows). ONL, outer nuclear layer; OPL, outer plexiform layer; INL, inner nuclear layer; IPL, inner plexiform layer; GCL, ganglion cell layer. Scale bar = 75 µm for C–D and 15 µm for E–G.

**Figure 2:**
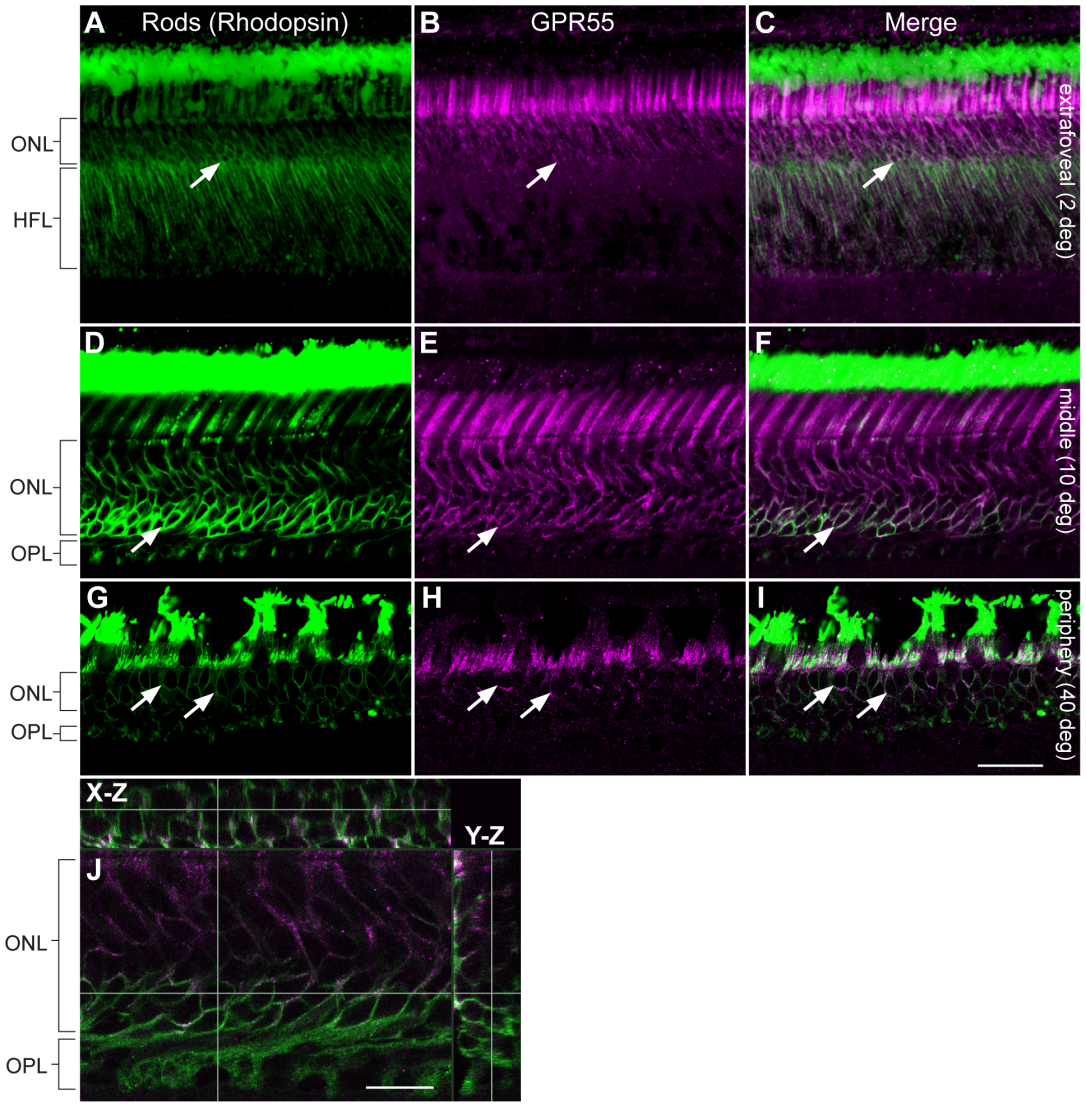
Double-label confocal immunofluorescence illustrating localization of rhodopsin and GPR55 in the monkey extrafoveal, middle, and peripheral retina. Rhodopsin-IR (green) was not restricted to the rod outer segments; however, this region had the most prominent staining. Note that GPR55-IR (magenta) is present throughout the rods, with the most prominent staining in the inner segments, and very faint staining in the outer segments and spherules. Arrows point to perinuclear staining. Rhodopsin-IR labeled rod photoreceptors in the monkey extrafoveal (**A–C**), middle (**D–F**), and peripheral retina (**G–I**), and these were GPR55 immunoreactive. Note that GPR55-IR is co-localized with rhodopsin-IR, and a 3D reconstruction in the X-Z and Y-Z axes showed no co-localization in rods nuclei (**J**). ONL, outer nuclear layer; HFL, Henle fiber layer; OPL, outer plexiform layer. Scale bar = 75 µm for A–C, 30 µm for D–I, and 15 µm for J.

**Figure 3:**
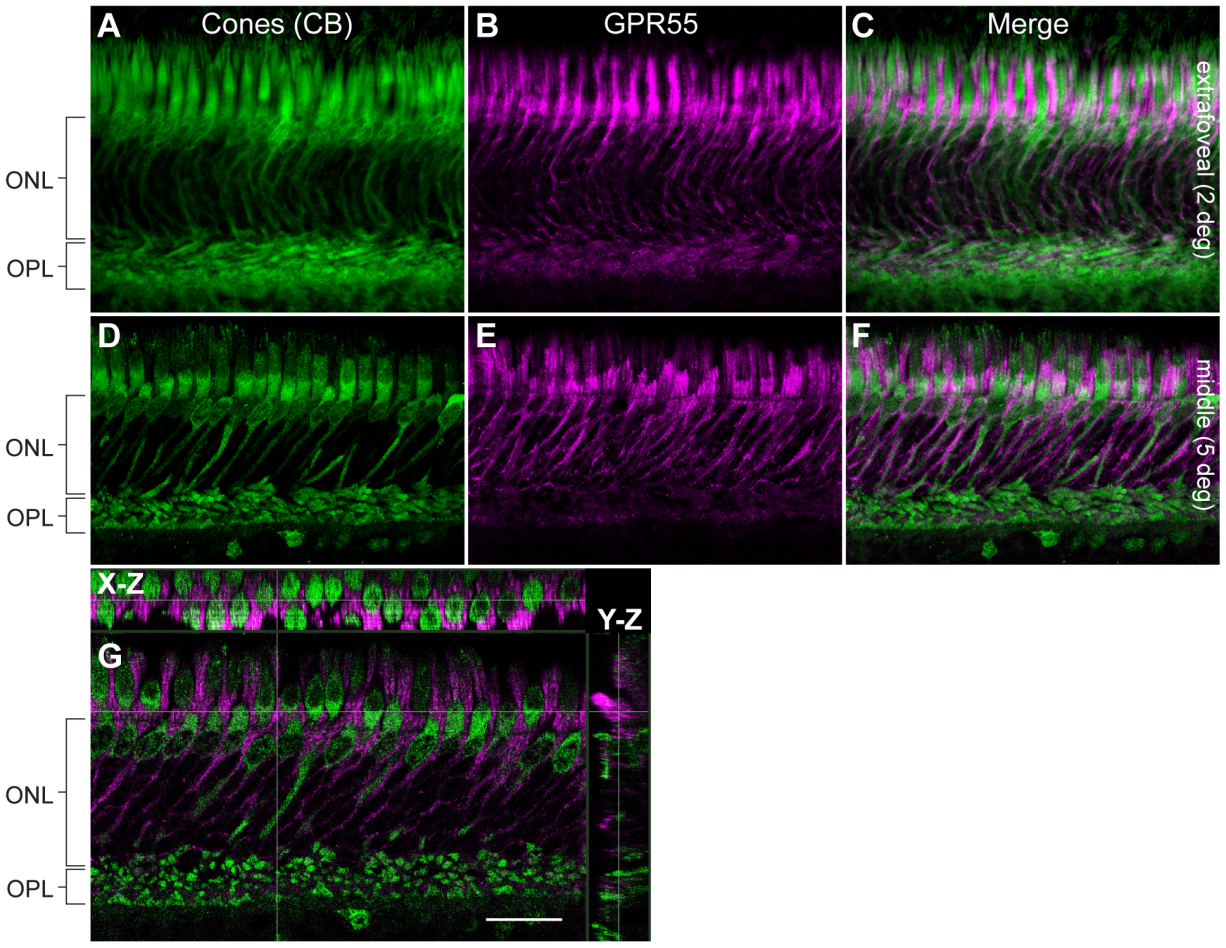
Double-label confocal immunofluorescence illustrating localization of calbindin (CB) and GPR55. Flattened Z-series showing CB-immunoreactive cones photoreceptors (green) in the monkey extrafoveal (**A–C**) and middle (**D–F**) retina, and these were not GPR55-immunoreactive (magenta). Note that GPR55-IR appears co-localized around the cones, but a 3D reconstruction in the X-Z and Y-Z axes showed no co-localization (**G**). This micrograph shows that the GPR55-immunoreactivity was adjacent to the CB-immunoreactive cone photoreceptors. ONL, outer nuclear layer; OPL, outer plexiform layer. Scale bar = 30 µm.

**Figure 4:**
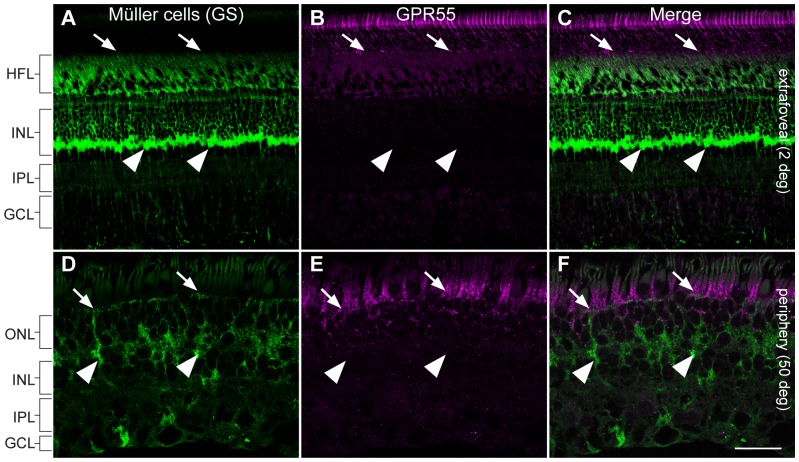
Double-label confocal immunofluorescence illustrating localization of glutamine synthetase (GS) and GPR55. GS-IR (green), labeling Müller cells in the primate retina, did not colocalize with GPR55-IR (magenta) in vertical sections taken from the extrafoveal region (**A–C**). Arrows indicate the projections of the Müller cell membrane in the apical margin known as apical villi that lack GPR55. Arrowheads point at Müller cell bodies that do not express GPR55. The absence of GPR55 and GS colocalization in Müller cells villi and cell bodies is also shown in vertical sections taken from the peripheral retina (**D–F**). HFL, Henle fiber layer; ONL, outer nuclear layer; INL, inner nuclear layer; IPL, inner plexiform layer; GCL, ganglion cell layer. Scale bar = 75 µm for A–C and 30 µm for D–F.

In order to determine GPR55 antibody specificity in the vervet monkey, we carried out immunoblots on homogenates of fresh vervet monkey retina (Figure 1A – lane 1), visual cortex (Figure 1A – lane 2), and cerebellum (Figure 1A – lane 3). The expected band was noted at 37 kDa for each type of homogenate. Additional protein signals were detected below 37 kDa in the visual cortex homogenate and above 37 kDa in the cerebellum homogenate, but pre-incubation with GPR55 blocking peptide completely abolished antibody signals (Figure 1B). The GAPDH antibody was used in the same blot to ensure the proper equalization and loading of all samples (Figure 1A and 1B, lower panels). As an added control, the GPR55 antibody was preadsorbed against its blocking peptide prior to incubation with retinal sections, resulting in an absence of staining signal in the section (Figure 1D). GPR55-immunoreactivity (IR) is present throughout the monkey retina, extending from the extrafoveal region to the periphery and densely expressed in rods (Figure 1C and Figure 2). GPR55 is absent in cones, and outer Müller glial cell processes (Figure 3 and Figure 4). While cone cell bodies are located in a single row right below the outer limiting membrane, rod cell bodies make up the rest of the ONL below the cone cell bodies. Careful examination of photoreceptors stained with Sytox green, a nuclear stain, and GPR55 indicates an absence of GPR55 expression in the nuclei of rods (Figure 1E–F, arrows).

### Cellular localization of GPR55 in the retina

The ONL is composed of cone and rod nuclei. There are also Müller cell outer processes that enroll these cell bodies. To distinguish cones and rods, double immunolabeling with cell specific markers was performed. Double-label of GPR55 with rhodopsin, a specific rod photoreceptor cell marker, allowed us to restrict the labeling to rod photoreceptors (Figure 2). Our results also show that GPR55 was not localized in cone photoreceptors of the monkey extrafoveal and middle retina (Figure 3). Dense GPR55 immunostaining was detected around the cone outer segments (Figure 3). High magnification of the PRL let us identify one cell population immunostained with GPR55 antibody. In the three pairs of retinas used for immunohistochemistry, we found the same staining pattern. No differences in the expression of GPR55 with regard to eccentricity were observed.

### No GPR55 expression in Müller cells

Double-labeling of GPR55 and GS, a specific marker for labeling both somata and processes of Müller cells [20], [21], was performed for assessing the expression of GPR55 in Müller cells. As shown in Figure 4, Müller cells span the entire neural retina and their processes expand in the GCL to form characteristic endfeet. No GPR55 immunoreactivity was detected in GS-positive somata and processes of Müller cells (Figure 4).

### No GPR55 expression in horizontal and bipolar cell extensions in the OPL

**Figure 5:**
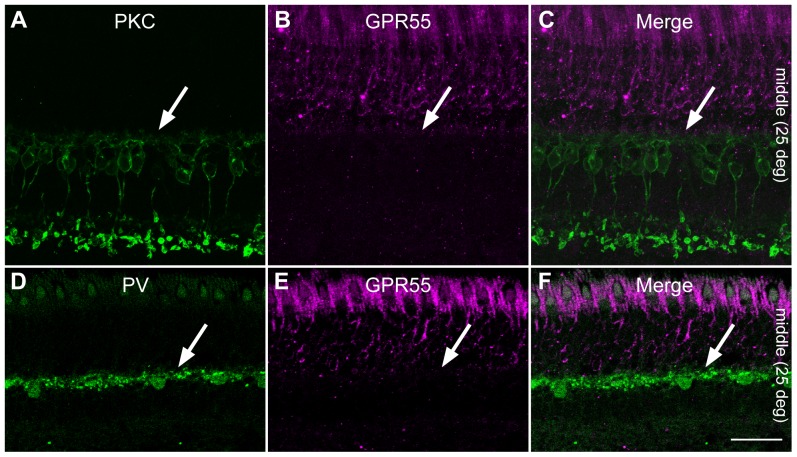
Double-label confocal immunofluorescence illustrating localization of PKC or parvalbumin (PV) and GPR55. PKC-IR (green), labeling specifically rod bipolar cells, did not colocalize with GPR55-IR (magenta) in vertical section taken from the middle retina (**A–C**). Additionally, PV-IR (green), marking horizontal cells in the primate retina, did not colocalize with GPR55-IR (magenta) vertical section taken from the middle retina (**D–F**). Scale bar = 30 µm for A–C and 75 µm for D–F.

We wanted to know if the little GPR55 expression found in rod spherules was also present in horizontal and bipolar cells outer processes. Double label of PKC, a rod bipolar cell marker, and GPR55 allowed us to establish that GPR55 was strictly localized in rods. Dendritic fibers of rod bipolar cells were rather juxtaposed to GRP55 immunoreactive rod spherules (Figure 5A–C). Moreover, GPR55 did not colocalize with parvalbumin, a marker that labels primate horizontal cells including their dendritic invaginations into rod photoreceptor terminals (Figure 5D–F).

### Labeling of GPR55, CB1R, and CB2R

**Figure 6:**
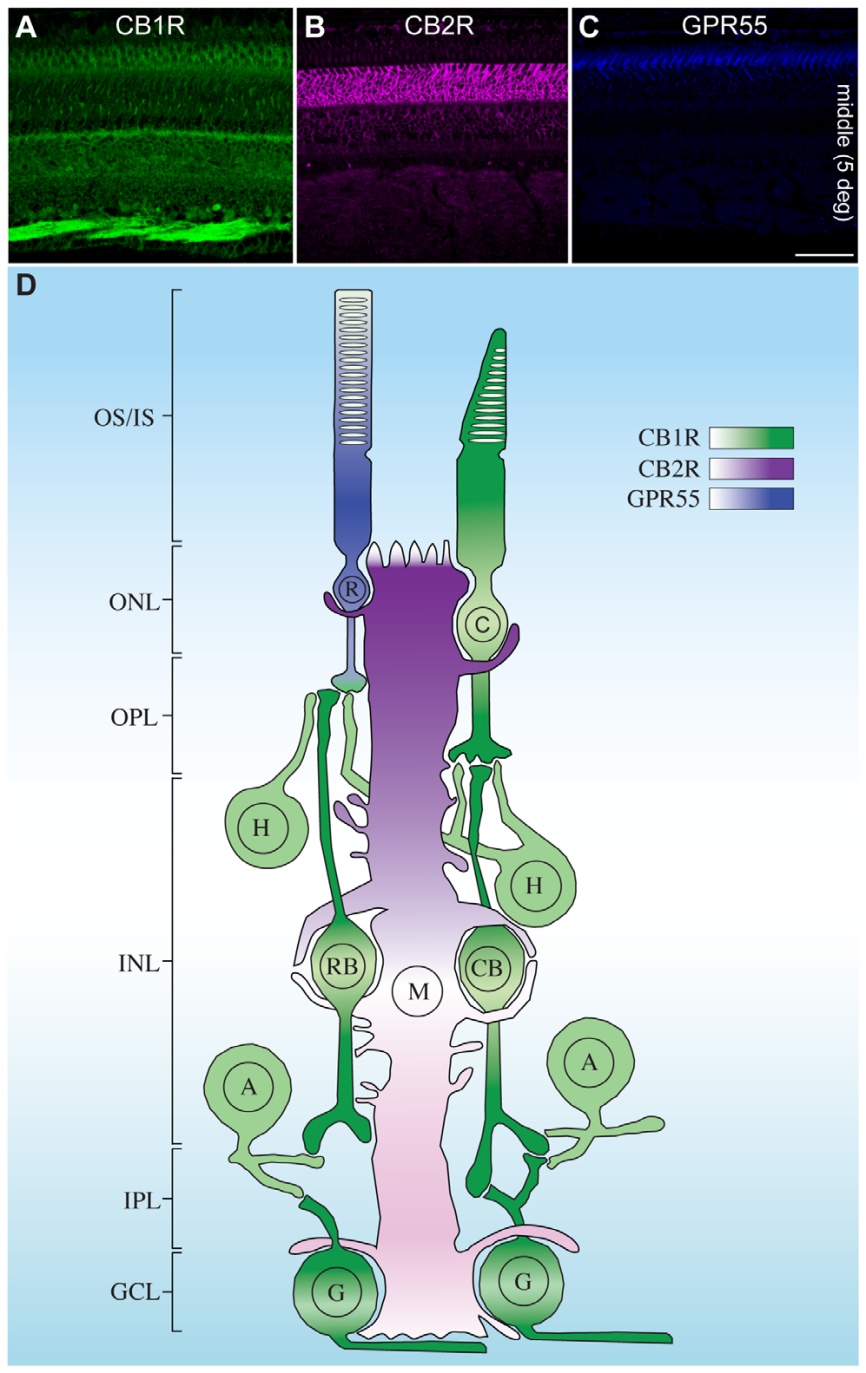
Confocal immunofluorescence images and a schematic illustration representing the localization of CB1R, CB2R, and GPR55 in the monkey retina. CB1R is localized in neural components, with very weak (albeit absence) of GPR55-IR in rods (**A**). CB2R is strictly expressed in the glial components, the Müller cells (**B**). GPR55 is found exclusively in rods, with the most prominent staining in the inner segments (**C**). Color bars in the schematic illustration (**D**) indicate the intensity of CB1R (green), CB2R (magenta), and GPR55 (blue) expressions. OS/IS, outer and inner segments of rods and cones; ONL, outer nuclear layer; OPL, outer plexiform layer; INL, inner nuclear layer; IPL, inner plexiform layer; GCL, ganglion cell layer; C, cones; R, rods; H, horizontal cells; RB, rod bipolar cells; CB, cone bipolar cells; A, amacrine cells; G, retinal ganglion cells; M, Müller cells. Scale bar = 75 µm.

The GPR55, CB1R, and CB2R antibodies that we selected came from the same host, making the use of simultaneous double-labeling protocol not adequate. We therefore used serial sections to compare the distribution of CB1R, CB2R, and GPR55 (Figure 6A–C). These 3 receptors are differentially expressed in the retina. CB1R is found in the neural retina, including the photoreceptors (cones with a prominent staining in their outer segments and pedicles, and rods with little staining restricted to their spherules), the horizontal cells, the bipolar cells, amacrine cells, and ganglion cells. CB2R is found in the glial component of the retina, namely Müller cells. GPR55 is exclusively expressed in rods, with a prominent signal in the inner segments. These data are summarized schematically in Figure 6D for all retinal cell types.

## Discussion

The present results demonstrate the existence of GPR55 in the monkey extrafoveal and peripheral retina. The localization of GPR55 in rods is important because, although the presence of CB1R and CB2R in the monkey retina is well established [20], [21], [44], we are still far from identifying the exact role of the cannabinoid receptors in primate retinal functions. The data presented here provide new information concerning cannabinoid receptors' expression in the monkey retina, and suggest new directions for uncovering their functions. It is somewhat surprising that protein from CB1R, CB2R, and GPR55 is detectable in distinct cell types of the monkey retina.

CB1R was localized in the neural components of the monkey central and peripheral retina [20]. CB1R immunoreactivity was present in cones, horizontal cells, bipolar cells, amacrine cells and ganglion cells. The most prominent expression of CB1R was found in the cones of the *fovea centralis*. The exact role of this receptor in retinal function is unknown although there is general agreement that cannabinoids suppress dopamine release and reduce neurotransmitter release from cones and bipolar cells [3].

CB2R neuronal expression has been ambiguous and controversial, but nevertheless, the majority agrees on the presence of this receptor in glial components of the CNS. Indeed, CB2R was detected in the glial component of the monkey retina, Müller cells [21]. The role of this receptor has been hypothesized and suggests that it is an important player for the regulation and buffering of potassium following light activation in the retina.

GPR55 expression was found specifically in rod photoreceptors and enriched in the inner segment, although smaller amounts of this protein could be detected essentially throughout the rods of the extrafoveal and peripheral regions of the retina. It is plausible that such an asymmetric distribution of GPR55 reflects its function in phototransduction in relation with transducin.

Given that CB1R is present in the neuroretina, CB2R in the retinal glia, and GPR55 in rods, we can hypothesize that each of these receptors has a unique retinal function. Furthermore, THC can bind to GPR55 and induce a signal transduction different from that of CB1R and CB2R [6]. Even though GPR55 is phylogenetically distinct from the traditional cannabinoid receptors, in some experimental paradigms, it is also activated by endocannabinoids, phytocannabinoids, and synthetic cannabinoid ligands [45]. While Kumar et al. (2012) using the same antibody reported the presence of GPR55 in human trabecular meshwork cells by Western blot analysis, these authors did not assess the localization of GPR55-positive cells in the retina. By double immunolabeling, we obtained a general picture of GPR55 localization in the monkey retina. A schematic diagram summarizing our results is presented in Figure 6. We report for the first time the presence of GPR55 in rod photoreceptors of the monkey retina. Certainly, immunohistochemistry is extremely sensitive and the presence of a small amount of protein does not guarantee a functionally important protein. It will be worthwhile to verify if CB1R-, CB2R-, or/and GPR55-blockade have an effect on retinal function.

### Hypothetical functional implications

**Figure 7:**
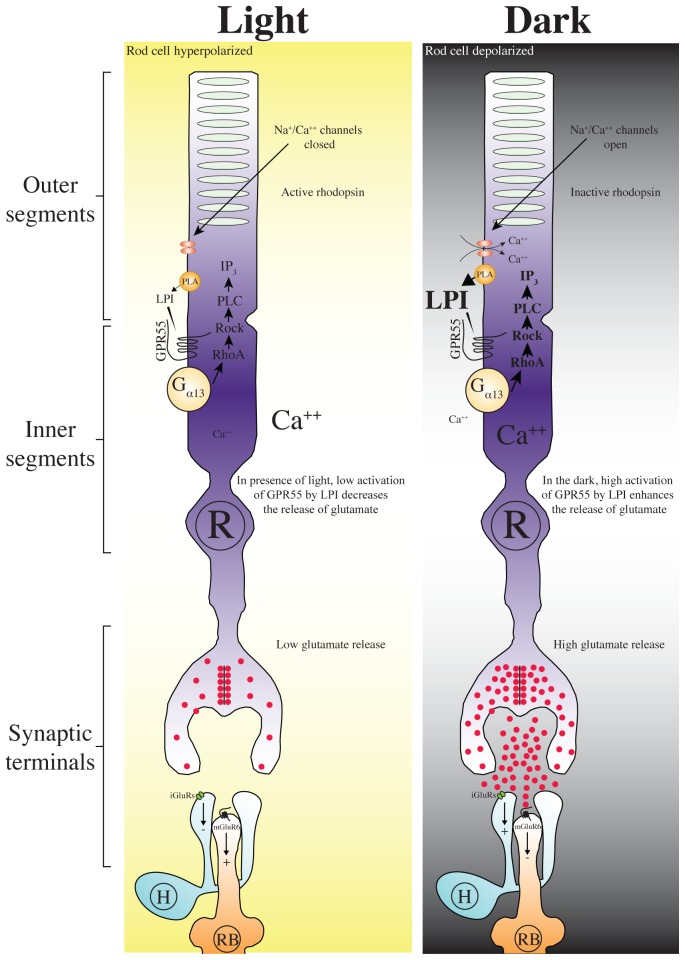
Schematic illustration depicting a hypothetical function for GPR55 in the monkey retina. Activation of GPR55 by LPI could represent a new modulation process in rods thus regulating scotopic vision. In the dark, rods are depolarized and a cytoplasmic isoform of phospholipase A2 can synthetize LPI and release it in the extracellular media. LPI binds GPR55 and activate distinct intracellular signaling cascades, including RhoA activation, IP_3_ release, and Ca^2+^ mobilization, ultimately controlling the release of glutamate. In presence of light, hyperpolarized rods produce low levels of LPI; in the dark, depolarized rods synthesize and accumulate high levels of LPI. R, rods; H, horizontal cells; RB, rod bipolar cells; LPI, lysophosphatidylinositol; iGluR, ionotropic glutamate receptors; mGluR, metabotropic glutamate receptors; cPLA, cytosolic phospholipase A2.

In the dark, rods are depolarized and constantly stimulated to allow the release of glutamate. Glutamate then binds to mGluR6 to hyperpolarize rod bipolar cells and to iGluRs to depolarize horizontal cells. It is well documented that LPI is a lysophospholipid-signaling molecule that modulates many cell functions. In fact, the generation of LPI is linked to the metabolism of membrane phospholipids by enzymes like phospholipase A1 and A2 that are activated upon cell stimulation and are located at the inner and outer side of the plasma membrane. Initially, LPI has been discussed to serve as second-messenger lysophospholipid that modulate intracellular signaling events [46]. LPI is a key signaling intermediate that modulates many aspects of cellular function. The identification of this ligand as a novel target for GPR55 suggests a fundamental role for this receptor in physiological processes. It has also been proposed that GPR55 exhibits a constitutive activity [47]. Characterization of the eCB system in other brain areas has shown that GPR55 activation induces intracellular Ca^++^ fluctuations through a RhoA-mediated, and inositol 1,4,5-trisphosphate (IP_3_)-sensitive mechanism mobilizing Ca^++^ stores [6], [8], [10]. In fact, stimulation of GPR55 by LPI evokes an intracellular Ca^2+^ rise in hippocampal slices [48]. We present here a hypothetical model of the role of GPR55 in retina. Given that rods are in a depolarized state and continuously active in the dark, a high tone of LPI activating GPR55, thereby leaving calcium channels open, maintains the constant release of glutamate. In the presence of light stimulation, rod membranes are hyperpolarized, LPI is in a lower basal tone, and the release of glutamate is reduced (Figure 7). We show here for the first time that GPR55 is exclusively expressed in rods of the vervet monkey, although further experiments are still needed in order to clarify its precise role in scotopic vision. In a companion study, we investigated the effects of intravitreal injections of LPI, a specific endogenous agonist of GPR55, on the scotopic electroretinogram of normal vervet monkeys. We found, in accordance with our hypothesis (illustrated in the model in Figure 7), that the scotopic ERG is modified by the activation of GPR55. Indeed, following the injection of LPI, there is a large increase in the rod response (unpublished data).
